# From need satisfaction to academic achievement: pathways through wellbeing and engagement among university students across different Italian institutions

**DOI:** 10.3389/fpsyg.2026.1937840

**Published:** 2026-10-15

**Authors:** Irene Florean, Micol Gemignani, Gianluca Mariano Colella, Valeria Verrastro, Francesca Liga, Cristiano Inguglia, Epifania Saputo, Sonia Grassini, Paola Venuti, Barbara Penolazzi, Giulio D'Urso, Francesco Craig

**Affiliations:** 1 Department of Life Sciences, University of Trieste, Trieste, Italy; 2 Department of Psychology and Cognitive Science, University of Trento, Rovereto, Italy; 3 Department of Cultures, Education and Society, University of Calabria, Rende, Italy; 4 Department of Health Sciences, Università degli Studi “Magna Graecia” di Catanzaro, Catanzaro, Italy; 5 Department of Clinical and Experimental Medicine, University of Messina, Messina, Italy; 6 Department of Psychology, Educational Science and Human Movement, University of Palermo, Palermo, Italy; 7 Conservatorio di Musica Alessandro Scarlatti, Palermo, Italy; 8 Department of Life Sciences, University of Siena, Siena, Italy; 9 Department of Law, Economics, and Human Sciences, Mediterranean University of Reggio Calabria, Reggio Calabria, Italy

**Keywords:** academic achievement, Positive Psychology, Self-Determination Theory, student engagement, student wellbeing

## Abstract

**Background:**

University students face personal and academic demands that may challenge their adaptive functioning. Although intrinsic goals, need satisfaction, wellbeing, and academic engagement have been distinctively linked to academic achievement, an integrated model specifying how these constructs are structurally related and associated with students' academic outcomes is lacking. The present study addresses this gap by testing models grounded in Self-Determination Theory and previous evidence.

**Methods:**

Participants were 2891 university students (*M*_*age*_ = 23.22 years, *SD*_*age*_ = 5.25 years; 74.75% women) recruited across multiple Italian universities. The online survey comprised different measures, including socio-demographic items, the Basic Psychological Need Satisfaction and Frustration Scale, the Aspiration Index, the SInAPSi Academic Engagement Scale, and the Mental Health Continuum-Short Form. Structural equation modeling was used to test two alternative model specifications. Starting from a more general model, progressively more parsimonious models were tested by constraining selected direct effects. The final model was selected based on comparative fit, overall model fit, parsimony, and theoretical coherence.

**Results:**

The retained model specified wellbeing as antecedent to academic engagement. Within this model, basic psychological need satisfaction showed indirect links to academic achievement through wellbeing and academic engagement, while intrinsic goals were related to achievement only through academic engagement. Academic engagement emerged as the most proximal predictor of achievement, mediating the pathways from need satisfaction, intrinsic goals, and wellbeing.

**Conclusions:**

These findings provide an integrated account of how satisfaction of basic psychological needs, intrinsic motivations, wellbeing, and academic engagement relate to academic achievement in university students. Our evidence supports a model in which psychological need satisfaction represents a relevant condition associated with wellbeing, intrinsic goals contribute more specifically to academic engagement, and academic engagement acts as the proximal mechanism linking these factors with academic achievement. Beyond advancing theoretical understanding, our results may inform preventive programs and interventions nurturing students' wellbeing while also promoting their academic performance.

## Introduction

In recent years, the mental health of university students has become a public concern worldwide. Mental health is conceptualized as a multidimensional construct, encompassing different aspects of wellbeing beyond the mere absence of psychopathology. The Mental Health Continuum model (Keyes, 2002) offers a framework integrating hedonic and eudaimonic components of wellbeing, while distinguishing among emotional, psychological, and social dimensions. Emotional wellbeing refers to positive affect and life satisfaction, psychological wellbeing to optimal personal functioning, including purpose in life, autonomy, self-acceptance, and personal growth, and social wellbeing to a sense of integration and positive functioning within the broader community. This model aligns with Positive Psychology (Ryff, 1989), which emphasizes the promotion of strengths, optimal functioning, and flourishing as key targets for both research and intervention in higher education settings (Ruini, 2022).

Evidence indicates that higher levels of psychological wellbeing are often associated with adaptive academic outcomes, including greater motivation, engagement, persistence, and academic adjustment (Antaramian, 2017; Howell, 2009). On the other hand, university students may experience substantial psychological difficulties, including distress, exhaustion, and diminished wellbeing, which can negatively relate to academic participation, commitment, and functioning (Bruffaerts et al., 2018; Emmerton et al., 2024). Data from the World Mental Health (WHO) Surveys indicate that one-third of university students screened positive for a DSM-IV disorder in the preceding year, pointing to a substantial burden of clinically relevant symptoms in this population (Auerbach et al., 2018). This scenario unfolds within a context characterized by increasingly pressing academic demands, ongoing structural transformations in higher education, uncertainty about future employment, and wider social and economic difficulties. For many students, academic and broader contextual pressures may overlap with the developmental challenges of emerging adulthood, including identity exploration, increasing autonomy, and multiple life transitions (Arnett, 2000; Sussman and Arnett, 2014; Mancinelli et al., 2026). Given this, the interplay of academic pressures, social dynamics, and developmental transitions could make the university years particularly relevant for mental health.

Among the empirically supported frameworks for explaining the psychological determinants of wellbeing, Self-Determination Theory (SDT; Deci and Ryan, 2000; Ryan and Deci, 2017) posits that human optimal functioning is rooted in the satisfaction of three basic psychological needs, namely autonomy, competence, and relatedness. Autonomy refers to the experience of volition and psychological freedom in one's actions; competence involves feeling effective, capable, and able to master challenges; and relatedness concerns the experience of mutual care, belonging, and social connection. These needs are conceived as universal and essential across different life domains, including education. Consistently, their satisfaction has emerged to be robustly correlated with wellbeing across different contexts (Van den Broeck et al., 2016). For instance, Eckley et al. (2023) provided longitudinal evidence supporting the role of basic psychological needs in adults' psychological wellbeing, showing that need satisfaction was associated with lower depressive and anxiety symptoms over time. Research conducted specifically with university students has similarly shown that satisfaction of autonomy, competence, and relatedness is positively associated with psychological wellbeing, academic engagement, and adaptive functioning, whereas need frustration is associated with maladaptive outcomes such as psychological distress, burnout, and internalizing symptoms (León and Núñez, 2013; Martela et al., 2023; Vansteenkiste and Ryan, 2013; Vansteenkiste et al., 2020). Chen et al. (2015) found that frustration of autonomy, competence, and relatedness was associated with greater depressive symptoms among university students, whereas satisfaction of these needs was linked to higher life satisfaction and vitality. Leow et al. (2023) additionally reported that greater basic psychological need satisfaction was associated with higher eudaimonic wellbeing among first-year university students, reflecting a stronger sense of optimal functioning and engagement in meaningful activities. Taken together, these findings suggest that students' wellbeing and adaptive academic functioning may be supported when their basic psychological needs are satisfied.

SDT also considers the content of the goals toward which individuals direct their efforts. While need satisfaction reflects psychological conditions that support wellbeing, aspirations provide direction to individuals' actions and motivational investment. Goal Content Theory (Kasser and Ryan, 1996) distinguishes between intrinsic aspirations, such as personal growth, close relationships, and community contribution, and extrinsic aspirations, such as financial success, fame, and image. Within the university context, intrinsic aspirations may shape how students interpret academic demands and invest effort in their studies; those aspirations are generally associated with more self-determined forms of motivation and greater wellbeing (Bradshaw et al., 2023; Davids et al., 2017). However, it has been suggested that the extent to which such aspirations translate into adaptive functioning partly depends on whether autonomy, competence, and relatedness are satisfied, thereby providing the psychological conditions required to pursue personally meaningful goals. Indeed, when basic psychological need satisfaction is considered, the unique contribution of intrinsic aspirations to wellbeing may substantially attenuate (Hope et al., 2019). Rather than indicating that intrinsic aspirations are irrelevant, this pattern points to differentiated but complementary roles within the SDT framework: basic psychological need satisfaction may represent a more proximal correlate of wellbeing, whereas intrinsic aspirations may capture a distinct aspect of motivational functioning related to the direction of students' goals and investment in their academic experiences. Considering both constructs therefore allows to examine how motivational orientations and psychological basic need satisfaction may jointly, but differently, relate to students' wellbeing and academic functioning.

Within higher education, motivational orientations and psychological wellbeing may influence how students engage with their academic environment, influencing the extent to which they invest effort, perceive meaning and value in their studies, and maintain commitment to their educational pathway. Academic engagement is typically conceptualized as a multidimensional construct encompassing behavioral, cognitive, and affective components, including active participation in learning activities, emotional involvement, and investment in understanding and mastering academic content (Fredricks et al., 2004). Evidence from recent studies supports the association between engagement, academic achievement, and wellbeing. In a longitudinal study, Datu and King (2018) showed that wellbeing predicted subsequent academic engagement among Filipino high school students. Similarly, a 3-year longitudinal study of Swiss students found reciprocal associations among wellbeing, school engagement, and academic achievement, suggesting that these constructs may reinforce one another over time (Schnell et al., 2025). Further longitudinal evidence indicates that students with higher psychological wellbeing are more likely to maintain engagement over time, particularly when academic demands increase (Salmela-Aro and Upadyaya, 2014; Van Wingerden et al., 2017). However, the literature on academic engagement is characterized by considerable conceptual heterogeneity, with several measures originally developed for non-university student populations. In the context of higher education, Freda et al. (2023) proposed a framework that emphasizes the complexity of academic engagement, stressing that engaged university students not only attend and participate in academic activities, but also sustain effort and commitment, self-regulate their choices, negotiate and share goals with others, and maintain a positive but realistic view of their study experience. To associate this theoretical conceptualization with a validated measure, the authors developed the SInAPSi Academic Engagement Scale (SAES), which identifies six dimensions of academic engagement. Three of these dimensions, such as perceived capability to persist in the university choice, university value and sense of belonging, and value of the university course, mainly capture students' motivational and value-related investment in their academic pathway. In the SAES validation study Freda et al., (2023), these dimensions showed meaningful associations with academic performance, supporting their relevance for understanding students' academic functioning.

It should be noted, however, that some other evidence has shown an opposite pattern of association between wellbeing and academic engagement. From a theoretical perspective, in fact, academic engagement may itself contribute to wellbeing, as sustained involvement in meaningful academic activities can provide students with experiences of competence, purpose, accomplishment, and social belonging. For example, Li et al. (2025) found that academic engagement partially mediated the relationship between basic psychological need satisfaction and psychological wellbeing in undergraduate students. Thus, rather than assuming a strictly linear pathway from wellbeing to engagement, the direction of this association should be considered an empirical question, with the possibility that wellbeing and engagement may reciprocally influence one another over time (Schnell et al., 2025).

Importantly, these processes can be relevant for students' academic achievement, which represents the main academic outcome considered in the present study. Academic achievement is inherently multifactorial, reflecting not only cognitive abilities and prior preparation, but also students' motivation, psychological functioning, and sustained engagement with their studies (Richardson et al., 2012; Robbins et al., 2004). In a meta-analysis, Richardson et al. (2012) identified several factors associated with academic achievement, with academic self-efficacy, self-set grade goals, and effort regulation, reflecting persistence and effort in challenging academic situations, showing medium-sized effects. Within the context of the present study, students whose basic psychological needs are more satisfied, who experience greater wellbeing, and who pursue more intrinsic and personally meaningful aspirations may be better positioned to perceive their academic activities as valuable, sustain effort, persist in their chosen study pathway, and remain involved when facing academic demands. Such sustained engagement may, in turn, represent a particularly relevant proximal correlate of academic performance.

Overall, this body of evidence suggests that academic engagement and wellbeing, together with motivational orientations and satisfaction of basic needs, can be considered important factors associated with students' academic functioning and achievement. However, the relationship between academic engagement and wellbeing remains unclear, with mixed evidence regarding the direction of their association. Moreover, although previous research has highlighted the importance of motivational orientations, basic psychological need satisfaction, and academic engagement for students' academic functioning, empirical literature has often examined these constructs separately (Bailey and Phillips, 2016; Baker, 2004). Consequently, there is still limited evidence clarifying how these factors are jointly associated within an integrated framework.

### Present research

Based on the empirical evidence reviewed above, we hypothesized that basic psychological need satisfaction and intrinsic aspirations would be positively associated with wellbeing, which in turn would be associated with greater academic engagement, and that academic engagement would be positively associated with academic achievement (Figure 1A). Given the mixed evidence concerning the relationship between wellbeing and academic engagement, we also tested an alternative theoretical ordering in which basic psychological need satisfaction and intrinsic aspirations were positioned as upstream correlates of academic engagement. Academic engagement was then specified as associated with wellbeing, which in turn was expected to be positively associated with academic achievement (Figure 1B).

**Figure 1:**
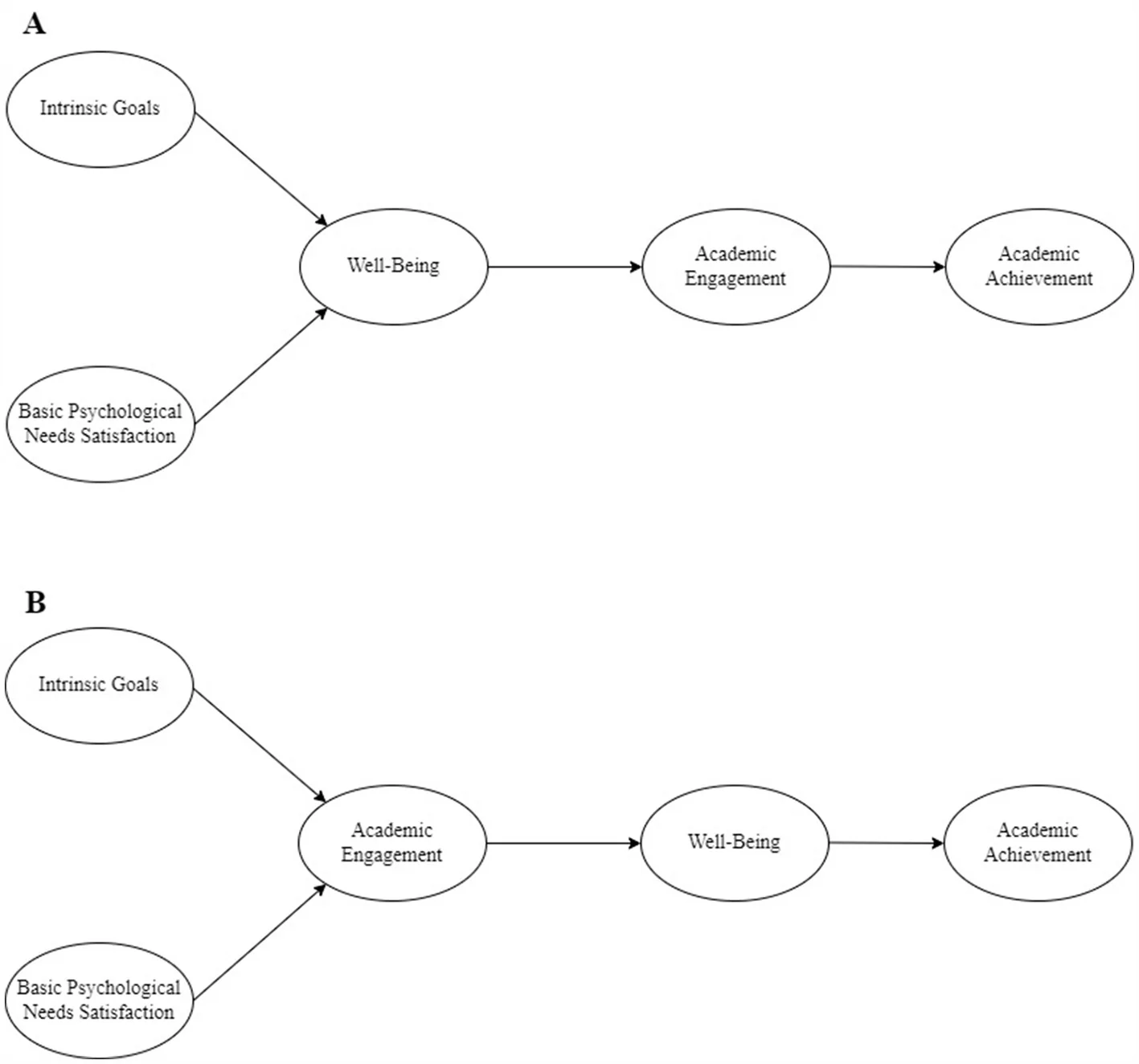
Primary and alternative hypothesized ordering among intrinsic goals, basic psychological need satisfaction, wellbeing, academic engagement, and academic achievement. **(A)** provides a schematic representation of the primary hypothesized ordering among the study variables, whereas **(B)** provides a schematic representation of the theoretically plausible alternative ordering tested in the study. Additional direct paths examined in the structural analyses are not displayed.

## Methods

### Participants

Participants were recruited across different Italian universities between March and October 2025. Eligibility criteria were being (a) at least 18 years old, (b) fluent in Italian, and (c) enrolled as a university or doctoral student. The initial dataset comprised 3,557 participants. Because the study focused on students enrolled in university degree programs (Bachelor's, Master's, or single-cycle programs) doctoral students (*n* = 261) and participants who did not report their degree-program enrollment (*n* = 7) were excluded. In addition, participants with missing responses on the exam-grade item (*n* = 278) or who explicitly indicated that they preferred not to respond to this item (*n* = 107) were not retained for subsequent analyses. As sex assigned at birth was included as a covariate in the main analyses, participants who preferred not to report their sex (*n* = 14) or had missing data (*n* = 1) were not included in the analytic sample. As responses in the intersex category were extremely rare (*n* = 1)^1^, analyses were conducted on participants reporting male or female sex categories. Applying these criteria, the final sample consisted of 2,891 university students (*M*_*age*_ = 23.22 years, *SD*_*age*_ = 5.25 years; range = 18–67 years), 730 men (25.25%) and 2,161 women (74.75%). In terms of gender identity, 2,687 participants (92.94%) identified as cisgender, 8 (0.28%) as transgender, and 22 (0.76%) as non-binary. An additional 66 participants (2.28%) selected the “Other” option, 38 (1.31%) preferred not to report their gender identity, and 70 (2.42%) had missing data on this item. Regarding degree program enrollment, 1,511 participants (52.27%) were enrolled in Bachelor's degree programs, 573 (19.82%) in Master's degree programs, and 807 (27.91%) in single-cycle degree programs. Demographic characteristics of the sample by study site are reported in Table 1. The study was conducted in accordance with the Declaration of Helsinki and its later amendments, as well as in compliance with the EU General Data Protection Regulation. All participants gave their consent before participating. The study was approved by the Ethical Committee (Prot. No. 0471730/2024) of the University of Calabria.

**Table 1:** Demographic characteristics of the sample by study site.

Variables	Catanzaro (*N* = 414)	Calabria (*N* = 429)	Messina (*N* = 413)	Palermo (conservatory) (*N* = 21)	Palermo (university) (*N* = 833)	Reggio Calabria (*N* = 151)	Siena (*N* = 207)	Trento (*N* = 180)	Trieste (*N* = 243)
Age	22.85 (4.32)	22.71 (5.41)	24.44 (6.34)	26.52 (8.74)	22.58 (3.92)	23.62 (6.14)	23.70 (5.38)	22.83 (5.10)	24.32 (6.65)
Sex assigned at birth									
Male	99 (23.91%)	115 (26.81%)	101 (24.46%)	8 (38.10%)	238 (28.57%)	10 (6.62%)	29 (14.01%)	63 (35%)	67 (27.57%)
Female	315 (76.09%)	314 (73.19%)	312 (75.54%)	13 (61.90%)	595 (71.43%)	141 (93.38%)	178 (85.99%)	117 (65%)	176 (72.43%)
Gender identity									
Cisgender	401 (96.86%)	389 (90.68%)	391 (94.67%)	19 (90.48%)	785 (94.24%)	131 (86.75%)	178 (85.99%)	173 (96.11%)	220 (90.53%)
Transgender	3 (0.72%)	0 (0.00%)	2 (0.48%)	0 (0.00%)	1 (0.12%)	0 (0.00%)	0 (0.00%)	0 (0.00%)	2 (0.82%)
Non-binary	1 (0.24%)	2 (0.47%)	1 (0.24%)	0 (0.00%)	8 (0.96%)	1 (0.66%)	1 (0.48%)	2 (1.11%)	6 (2.47%)
Other	9 (2.17%)	0 (0.00%)	7 (1.69%)	0 (0.00%)	39 (4.68%)	0 (0.00%)	5 (2.42%)	2 (1.11%)	4 (1.65%)
Prefer not to indicate	0 (0.00%)	12 (2.80%)	12 (2.91%)	2 (9.52%)	0 (0.00%)	3 (1.99%)	0 (0.00%)	2 (1.11%)	7 (2.88%)
Missing	0 (0.00%)	26 (6.06%)	0 (0.00%)	0 (0.00%)	0 (0.00%)	16 (10.60%)	23 (11.11%)	1 (0.56%)	4 (1.65%)
University degree program									
Bachelor	173 (41.79%)	169 (39.39%)	224 (54.24%)	12 (57.14%)	509 (61.10%)	62 (41.06%)	116 (56.04%)	101 (56.11%)	145 (59.67%)
Master	67 (16.18%)	64 (14.92%)	62 (15.01%)	9 (42.86%)	202 (24.25%)	24 (15.89%)	36 (17.39%)	54 (30.00%)	55 (22.63%)
Single-cycle	174 (42.03%)	196 (45.69%)	127 (30.75%)	0 (0.00%)	122 (14.65%)	65 (43.05%)	55 (26.57%)	25 (13.89%)	43 (17.70%)

Values are *M* (*SD*) for age and *n* (%) for categorical variables.

### Procedure

The present research is embedded within an Italian national project (PRO-BENE-COMUNE), a large-scale initiative aimed at promoting wellbeing among university students through multiple preventive and promotional actions. Within this broader framework, the multicenter research^2^ reported here investigated the associations between individual and motivational characteristics (basic psychological needs, personal aspirations), wellbeing, academic engagement and performance indicators, while also investigating other relevant constructs in the population of university students. The recruitment across different universities was primarily conducted through a convenience sampling method. In total, 10 universities located in Northern, Central, and Southern Italy, as well as in the Islands, were involved in the study. The survey link was distributed via different online platforms (e.g., Qualtrics, Google Forms) and shared through institutional media of each university. Participants were generally encouraged to share the survey link with other university students, incorporating elements of snowball sampling into the recruitment strategy. The survey assessed the study variables in the same order in which they are presented in the Measures section.

### Measures

A complete list of items and response scales is provided in the Supplementary Section S4, and Supplementary Table S12.

#### Socio-demographic data

Socio-demographic items covered information like age, sex assigned at birth, gender identity, degree course, nationality, relationship status, year of enrollment in the degree program, living situation, commuting status, and the presence (or absence) of a certified disability.

#### Basic psychological satisfaction and frustration scale (BPNSFS)

The *Basic Psychological Need Satisfaction and Frustration Scale* (BPNSFS; Chen et al., 2015; Italian version validated by Costa et al., 2018) is a self-report questionnaire assessing satisfaction and frustration of the three basic psychological needs central to SDT (Deci and Ryan, 2000; Ryan and Deci, 2017), that is *autonomy* (feeling in control of one's actions), *competence* (feeling effective and capable), and *relatedness* (feeling connected to others). The full scale (12 items) comprises six subscales, corresponding to satisfaction and frustration of each need: autonomy satisfaction, autonomy frustration, competence satisfaction, competence frustration, relatedness satisfaction, and relatedness frustration. Items are rated on a 5-point Likert scale ranging from 1 (not at all) to 5 (a lot). Sample items include “I feel a sense of choice and freedom in the things I commit to” and “I feel excluded from the group I want to belong to”. Although the instrument provides scores for all six subscales, the present study focused specifically on the satisfaction dimension. Therefore, only the three satisfaction subscales, namely autonomy satisfaction, competence satisfaction, and relatedness satisfaction, were combined to compute an overall Basic Psychological Need Satisfaction score (BPN-S), according to prior evidence (Liga et al., 2020). Higher scores indicated greater overall satisfaction of basic psychological needs. The internal consistency of the BPN-S was satisfactory (McDonald ω = 0.76).

#### Aspiration index (AI)

The *Aspiration Index* (Kasser and Ryan, 1996; Duriez et al., 2007) was used to assess life aspiration orientation. Using the 12-item version of the scale (Barberis et al., 2024; Duriez et al., 2007), participants indicated the priority attributed to each aspiration on a 5-point Likert scale ranging from 1 (never) to 5 (always). The measure includes six items assessing *Intrinsic Goals* (IG), with two items each referring to self-acceptance, community feeling, and affiliation, and six items assessing *Extrinsic Goals* (EG), with two items each referring to financial success, social recognition, and attractive appearance. For the two dimensions, sample items are “It is important for me to do something that contributes to improving society” or “It is important for me to be recognized and admired for the things I do”. Given its established associations with wellbeing and positive psychological functioning, the present analyses focused on the IG subscale. The internal consistency of the scale was satisfactory (McDonald ω = 0.78).

#### SInAPSi academic engagement scale (SAES)

The *SInAPSi Academic Engagement Scale* (SAES; Freda et al., 2023) consists of 29 items rated on a 5-point Likert scale ranging from 1 (not at all) to 5 (totally). The instrument operationalizes academic engagement across six dimensions: (1) *perceived ability to persist in the chosen university pathway* (e.g., “I would leave the university right away if I had an alternative”); (2) *perceived value of the university and sense of belonging* (e.g., “Attending university is a great opportunity for me”); (3) *perceived value of the university course* (e.g., “I find my studies very meaningful for my professional plans”); (4) *relationships between the university and one's relational network* (e.g., “I talk about my professional plans with my family”); (5) *engagement with university peers* (e.g., “Studying with other students is useful to me”); and (6) *engagement with university professors* (e.g., “My professors are interested in my opinions and what I say”). The SAES has demonstrated a valid factor structure and good convergent, discriminant, construct-related, and criterion-related validity (Freda et al., 2023). Given their established associations with academic outcomes, we used the first three subscales of the measure, which showed very good internal consistency (*AE-Persistence in Study Choice*, McDonald ω = 0.79; *AE-University Value & Belonging*, McDonald ω = 0.86; *AE-University Course Value*, McDonald ω = 0.91).

#### Mental health continuum–short form (MHC-SF)

The *Mental Health Continuum-Short Form* (MHC-SF; Keyes, 2007) is a 14-item self-report measure assessing positive mental health across three dimensions, such as emotional, psychological, and social wellbeing. Sample items include “How often did you feel interested in life?,” as well as items referring to feeling happy, satisfied with life, and confident. Participants reported the frequency of these experiences over the past month using a 6-point scale ranging from 0 (never) to 5 (every day). In addition to the total (composite) score (*Overall WB*), subscale scores can be computed for emotional, psychological, and social wellbeing. In the present study, internal consistency of each of these scales was good (*E-WB*, McDonald ω = 0.87; *S-WB*, McDonald ω = 0.84; *P-WB*, McDonald ω = 0.88; *Overall WB*, McDonald ω = 0.94).

#### Academic achievement

Academic achievement was assessed through students' self-reported average exam grade. Specifically, participants were asked “What is the overall average of your exam grades in your current degree program?”. Response options were “18–21”, “22–25”, “26–28”, “29–30,” “prefer not to answer,” and “not applicable.” The “not applicable” option was intended for doctoral students, who do not typically have an average exam grade. These options covered the full range of the Italian university grading system while allowing participants to refrain from responding if they did not wish to do so. Consistent with its measurement as ordered grade categories, the variable was treated as an ordinal indicator in the analyses.

#### Data analysis

All analyses were conducted in R within RStudio (version 4.4.3). Descriptive statistics and zero-order correlations were computed to summarize the study variables and their bivariate associations.

Prior to structural modeling, preliminary confirmatory factor analyses (CFA) were conducted to evaluate the measurement properties of the instruments and to assess whether the planned level of construct aggregation for the structural equation models (SEMs) was empirically supported. Because instrument items were measured on ordinal Likert-type scales, CFA models were estimated using the weighted least squares mean- and variance-adjusted estimator (WLSMV), which is recommended for ordered-categorical indicators (Li, 2016). Missing data in CFAs were handled using complete-case (listwise) deletion. Given the very low proportion of missing responses, complete-case estimation was unlikely to meaningfully affect parameter estimates while ensuring that all model-implied statistics were based on a consistent sample (Schafer and Graham, 2002). Model fit was evaluated using the Comparative Fit Index (CFI), Tucker-Lewis Index (TLI), Root Mean Square Error of Approximation (RMSEA), and Standardized Root Mean Square Residual (SRMR) (Hu and Bentler, 1999; Shi et al., 2018). Because RMSEA can be inflated in models with few degrees of freedom and when estimated using categorical estimators, interpretation primarily considered CFI and TLI together with standardized factor loadings, whereas RMSEA and SRMR are reported for completeness (Kenny et al., 2015; Li, 2016; Shi et al., 2018). Composite reliability (CR) and average variance extracted (AVE) were also computed to evaluate internal consistency and convergent validity. Full CFA results, including fit indices, standardized loadings, and reliability estimates, are reported in the Supplementary Section S2.

In the SEMs, Intrinsic Goals (IG) and Basic Psychological Need Satisfaction (BPN-S) were represented as single-indicator latent variables using their respective scale mean scores, with factor loadings fixed to 1. To account for measurement error, the residual variance of each indicator was fixed to (1–CR) x Var(X) and the corresponding latent variance to CR x Var(X) (Kline, 2023), based on the composite reliability estimate obtained from the corresponding item-level CFA. IG and BPN-S were allowed to covary. Academic Engagement (AE) was specified as a latent construct indicated by the aggregated scores for persistence in study choice, university value and sense of belonging, and university course value. Wellbeing (WB) was similarly specified as a latent construct indicated by Emotional, Social, and Psychological wellbeing.

The primary theoretical ordering positioned IG and BPN-S as upstream predictors of WB, followed by AE and academic achievement. Academic achievement was operationalized as mean exam grade and entered as a four-level ordered-categorical outcome. Age and sex assigned at birth (0 = male, 1 = female) were included as covariates predicting both mediators (i.e., WB and AE) and the outcome. Because the outcome variable was ordinal, SEMs were estimated using WLSMV, which accommodates categorical endogenous variables through threshold modeling within a latent response variable framework (Li, 2016; Xia and Yang, 2019). Indirect effects were evaluated using the delta method (Rosseel, 2012; Sobel, 1982). Missing data in SEM were handled using complete-case (listwise) deletion, ensuring that parameters were estimated on a consistent sample and avoiding data imputation.

To evaluate the robustness of the hypothesized structural relations, we followed a theory-guided, general-to-specific approach (Kline, 2023; West et al., 2012). Starting from a more general model, progressively more parsimonious versions were tested by constraining selected direct effects. For the primary WB → AE model specification, we first estimated the most general model, which included all theoretically plausible direct and indirect paths among the constructs. We then evaluated progressively more parsimonious models by removing selected direct paths. Successive models that differed only in the imposed path constraints were compared using the scaled (shifted) chi-square difference test appropriate for WLSMV estimation (Satorra, 2000), in conjunction with global fit indices, parsimony, and theoretical interpretability. This approach was adopted to assess whether additional direct effects significantly improved model fit, or whether a more parsimonious model adequately accounted for the observed associations.

The theory-driven alternative model specification was examined by reversing the specified position of WB and AE. In this AE → WB ordering, IG and BPN-S were positioned as upstream predictors of AE, followed by WB and academic achievement. The same general-to-specific strategy was used to evaluate progressively more parsimonious specifications differing in the direct paths retained. Because the best-fitting models representing the WB → AE and AE → WB specifications imposed different ordering structures, their comparison relied on descriptive evaluation of global fit, explained variance, parsimony, and theoretical coherence rather than chi-square difference testing (West et al., 2012). Both CFA and SEM models were estimated using the lavaan package (Rosseel, 2012). Full model specifications and within and between model comparisons are reported in the Supplementary Section S3.

## Results

### Preliminary analyses

Table 2 reports descriptive statistics for the study variables in the total sample and separately by study site.^3^ Figure 2 presents zero-order correlations among variables. Intrinsic goals (IG) and basic psychological need satisfaction (BPN-S) were positively associated with overall wellbeing and academic engagement, with associations generally stronger for BPN-S. Students who endorsed more intrinsic goals, as well as those who felt more autonomous in their choices, more competent in their academic activities, and more connected to others, reported higher wellbeing and greater academic engagement. Academic engagement, particularly persistence in study choice, showed a small positive association with academic achievement. Students who were more determined to continue along their chosen university path tended to obtain slightly higher mean exam grades. Overall, the pattern of correlations was consistent with the hypothesized models, supporting the use of SEM.

**Table 2:** Descriptive statistics for study variables by study site and in the total sample.

Variables	Catanzaro (*N* = 414)	Calabria (*N* = 429)	Messina (*N* = 413)	Palermo (conservatory) (*N* = 21)	Palermo (university) (*N* = 833)	Reggio Calabria (*N* = 151)	Siena (*N* = 207)	Trento (*N* = 180)	Trieste (*N* = 243)	Total sample (*N* = 2891)
IG	4.57 (0.50)	4.51 (0.52)	4.59 (0.49)	4.15 (0.41)	4.56 (0.45)	4.66 (0.49)	4.54 (0.50)	4.46 (0.45)	4.50 (0.48)	4.55 (0.48)
BPN-S	3.93 (0.65)	3.92 (0.63)	3.91 (0.68)	2.84 (0.49)	3.99 (0.57)	4.10 (0.59)	3.92 (0.66)	3.78 (0.61)	3.74 (0.63)	3.92 (0.63)
AE—persistence in study choice	4.08 (0.87)	4.03 (0.86)	4.00 (0.90)	3.88 (0.77)	3.91 (0.88)	3.93 (0.94)	3.75 (1.04)	4.02 (0.80)	3.84 (0.99)	3.96 (0.90)
AE—university value and sense of belonging	4.51 (0.55)	4.48 (0.58)	4.48 (0.59)	4.36 (0.59)	4.27 (0.66)	4.49 (0.63)	4.41 (0.65)	4.38 (0.59)	4.29 (0.66)	4.40 (0.62)
AE—university course value	4.31 (0.76)	4.41 (0.67)	4.36 (0.68)	4.15 (0.59)	4.21 (0.70)	4.46 (0.66)	4.22 (0.76)	4.18 (0.72)	4.13 (0.78)	4.28 (0.72)
Overall WB	2.65 (0.97)	2.62 (0.99)	2.57 (0.99)	2.96 (0.65)	2.61 (0.93)	2.86 (1.02)	2.54 (1.08)	2.51 (0.90)	2.45 (0.97)	2.60 (0.97)
E-WB	2.89 (1.10)	2.92 (1.13)	2.89 (1.16)	3.46 (0.64)	2.92 (1.05)	3.09 (1.22)	2.93 (1.29)	2.95 (1.05)	2.91 (1.15)	2.93 (1.12)
P-WB	3.10 (1.09)	3.03 (1.11)	3.13 (1.12)	3.33 (0.76)	3.04 (1.08)	3.39 (1.11)	3.05 (1.23)	2.93 (1.11)	2.83 (1.09)	3.06 (1.11)
S-WB	1.96 (1.07)	1.91 (1.09)	1.70 (1.11)	2.08 (0.92)	1.88 (1.07)	2.11 (1.19)	1.75 (1.09)	1.64 (0.95)	1.61 (1.00)	1.84 (1.08)
Mean exam grade										
18–21	8 (1.93%)	21 (4.90%)	7 (1.69%)	0 (0.00%)	23 (2.76%)	6 (3.97%)	7 (3.38%)	7 (3.89%)	8 (3.29%)	87 (3.01%)
22–25	147 (35.51%)	177 (41.26%)	114 (27.60%)	3 (14.29%)	207 (24.85%)	26 (17.22%)	58 (28.02%)	47 (26.11%)	62 (25.51%)	841 (29.09%)
26–28	200 (48.31%)	182 (42.42%)	206 (49.88%)	10 (47.62%)	423 (50.78%)	83 (54.97%)	91 (43.96%)	87 (48.33%)	123 (50.62%)	1405 (48.60%)
29–30	59 (14.25%)	49 (11.42%)	86 (20.82%)	8 (38.10%)	180 (21.61%)	36 (23.84%)	51 (24.64%)	39 (21.67%)	50 (20.58%)	558 (19.30%)

Values are *M* (*SD*) for continuous variables and *n* (%) for mean exam-grade categories. IG, Intrinsic Goals; BPN-S, Basic Psychological Need Satisfaction; AE, Academic Engagement; Overall WB, Overall WellBeing; E-WB, Emotional WellBeing; P-WB, Psychological WellBeing; S-WB, Social WellBeing.

**Figure 2:**
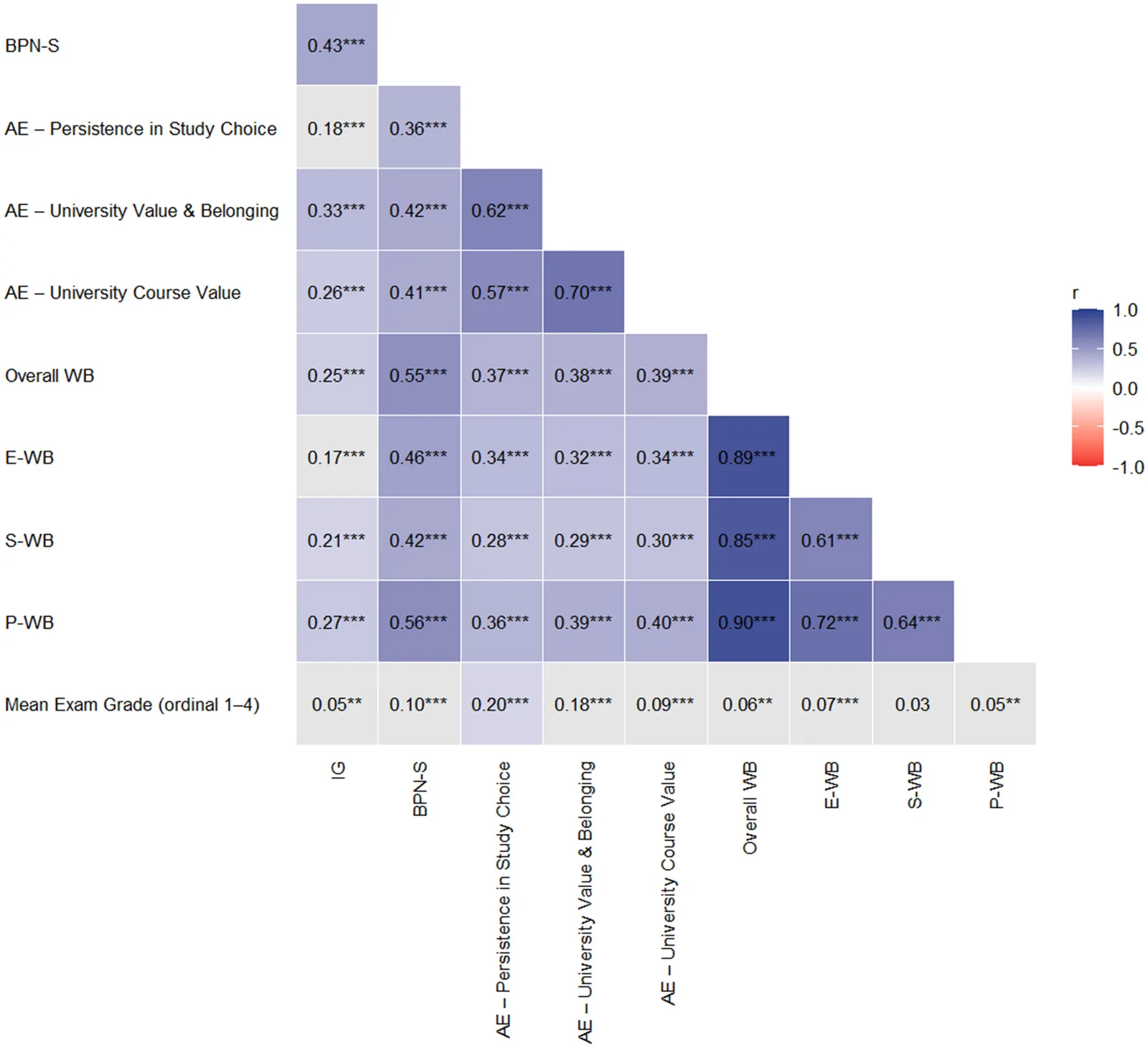
Correlation heatmap between study variables. Tiles are color-coded only for correlations of practical relevance (|*r*| ≥ 0.20); correlations below this threshold are shown in gray. IG, Intrinsic Goals; BPN-S, Basic Psychological Need Satisfaction; AE, Academic Engagement; Overall WB, Overall Well-Being; E-WB, Emotional WellBeing; S-WB, Social WellBeing; P-WB, Psychological WellBeing. Correlations were calculated using pairwise-complete observations, with sample sizes ranging from *N* = 2869 to *N* = 2889. ***p < 0.001; **p < 0.01.

### Structural equation modeling

Based on prior evidence suggesting that wellbeing (WB) and academic engagement (AE) may reciprocally influence one another, two structural model specifications differing in the specified ordering of WB and AE (WB → AE vs. AE → WB) were tested.

### Primary model specification

Within the WB → AE model specification, the model allowing intrinsic goals (IG) and basic psychological need satisfaction (BPN-S) to be directly linked to both WB and AE, WB to be linked to AE, and AE to be directly linked to mean exam grade (i.e., the WB → AE partially mediated model) provided the best-supported specification. This model did not lead to a significant deterioration in model fit relative to the least constrained model and was therefore preferred on grounds of parsimony (Supplementary Section S2).

### Alternative model specification

Within the AE → WB model specification, the model in which all constructs, namely IG, BPN-S, AE, and WB, were directly linked to mean exam grade (i.e., the AE → WB direct-effects model) emerged as the best-fitting specification, whereas more restrictive mediated structures resulted in a significant deterioration in model fit (Supplementary Section S3).

### Comparison between the two model specifications

When comparing the two model specifications based on their best-fitting models, the WB → AE partially mediated model demonstrated greater parsimony while maintaining comparable model fit and explanatory power for academic achievement relative to the AE → WB direct-effects model. Accordingly, the partially mediated model from the WB → AE specification was retained as the final model.

In the retained model, BPN-S showed a strong positive association with WB, β = 0.68, 95% CI [0.62, 0.73], *p* < 0.001, whereas IG showed a very small negative association with WB, β = −0.05, 95% CI [−0.10, 0.00], *p* = 0.043. Both IG, β = 0.11, 95% CI [0.06, 0.15], *p* < 0.001, and BPN-S, β = 0.30, 95% CI [0.23, 0.36], *p* < 0.001, positively predicted AE, and WB was also positively associated with AE, β = 0.30, [0.24, 0.35], *p* < 0.001. In turn, AE predicted mean exam grade, β = 0.18, 95% CI [0.14, 0.22], *p* < 0.001.

Focusing on indirect effects, IG showed a positive indirect association with mean exam grade via AE, β = 0.019, 95% CI [0.010, 0.027], *p* < 0.001, but also a very small negative indirect association through the serial pathway WB → AE, β = −0.003, 95% CI [−0.005, 0.000], *p* = 0.045. BPN-S was positively associated with mean exam grade both through AE, β = 0.053, 95% CI [0.036, 0.070], *p* < 0.001, and through the serial pathway WB → AE, β = 0.036, 95%CI [0.025, 0.046], *p* < 0.001. Finally, WB was positively associated with academic achievement through AE, β = 0.053, 95% CI [0.037, 0.069], *p* < 0.001.

It should be noted that all structural relations in the retained model were consistent with the hypothesized framework, with one exception. Specifically, IG showed a very small negative association with WB, and a very small negative indirect association with mean exam grade through the serial pathway WB → AE. However, these associations emerged in a structure where IG was allowed to covary with BPN-S, a highly correlated construct (*r* = 0.50). Consistent with our framework and previous literature, preliminary analyses showed positive, although modest, associations between IG and all WB dimensions, ranging from *r* = 0.17 (E-WB) to *r* = 0.27 (P-WB).

### Follow-up examination of the IG-WB association

To formally examine whether IG was negatively associated with WB once BPN-S was taken into account, a hierarchical regression analysis was conducted using the observed composite score of WB^4^. When considered alone, IG showed a significant positive association with WB (β = 0.24, *p* < 0.001). However, when BPN-S was included in the model, the association between IG and WB was no longer significant (β = 0.01, *p* = 0.619), whereas BPN-S remained significantly associated (β = 0.54, *p* < 0.001). The same pattern emerged when age and sex were included as control variables; that is, BPN-S remained significantly associated with WB (β = 0.54, *p* < 0.001), whereas IG continued to be non-significant (β = 0.02, *p* = 0.324).

Considering this pattern, we tested a more parsimonious model (hereafter the WB → AE constrained model), which was identical to the WB → AE partially mediated model, except for the path linking IG to WB, which was constrained to zero. Model fit for the WB → AE partially mediated and the WB → AE constrained models are reported in Table 3.

**Table 3:** Model fit indices and model comparison for the wellbeing → academic engagement partially mediated and constrained models.

Model	χ^2^	df	CFI	TLI	RMSEA	SRMR	Δχ^2^	Δdf	*p*
WB → AE partially mediated	152.07	37	0.95	0.96	0.05	0.02			
WB → AE constrained	155.35	38	0.95	0.96	0.05	0.03	4.40	1	0.036

CFI, Comparative Fit Index; TLI, Tucker-Lewis Index; RMSEA, Root Mean Square Error of Approximation; SRMR, Standardized Root Mean Square Residual. Reported CFI/TLI/RMSEA correspond to the robust values provided under WLSMV estimation.

Although the WB → AE constrained model showed a statistically significant deterioration in model fit compared to the WB → AE partially mediated model, global fit indices remained substantially unchanged across the two models (Table 2). Given that the path linking IG to WB was extremely small in magnitude and its removal did not meaningfully affect overall model fit, the constrained specification was retained as the final model for reasons of parsimony and interpretability. Model comparisons further indicated that the constrained model did not significantly worsen model fit relative to the least constrained specification within the WB → AE family (Δχ^2^(4) = 9.07, *p* = 0.059), while remaining substantially better in fitting data compared to the most constrained model (Δχ^2^(1) = 304.03, *p* < 0.001). See Supplementary Section S2 for the full set of model specifications. Figure 3 provides graphical representations of the best-supported model within each structural specification. Table 4 reports the indirect effects estimated in the final constrained model.

**Figure 3:**
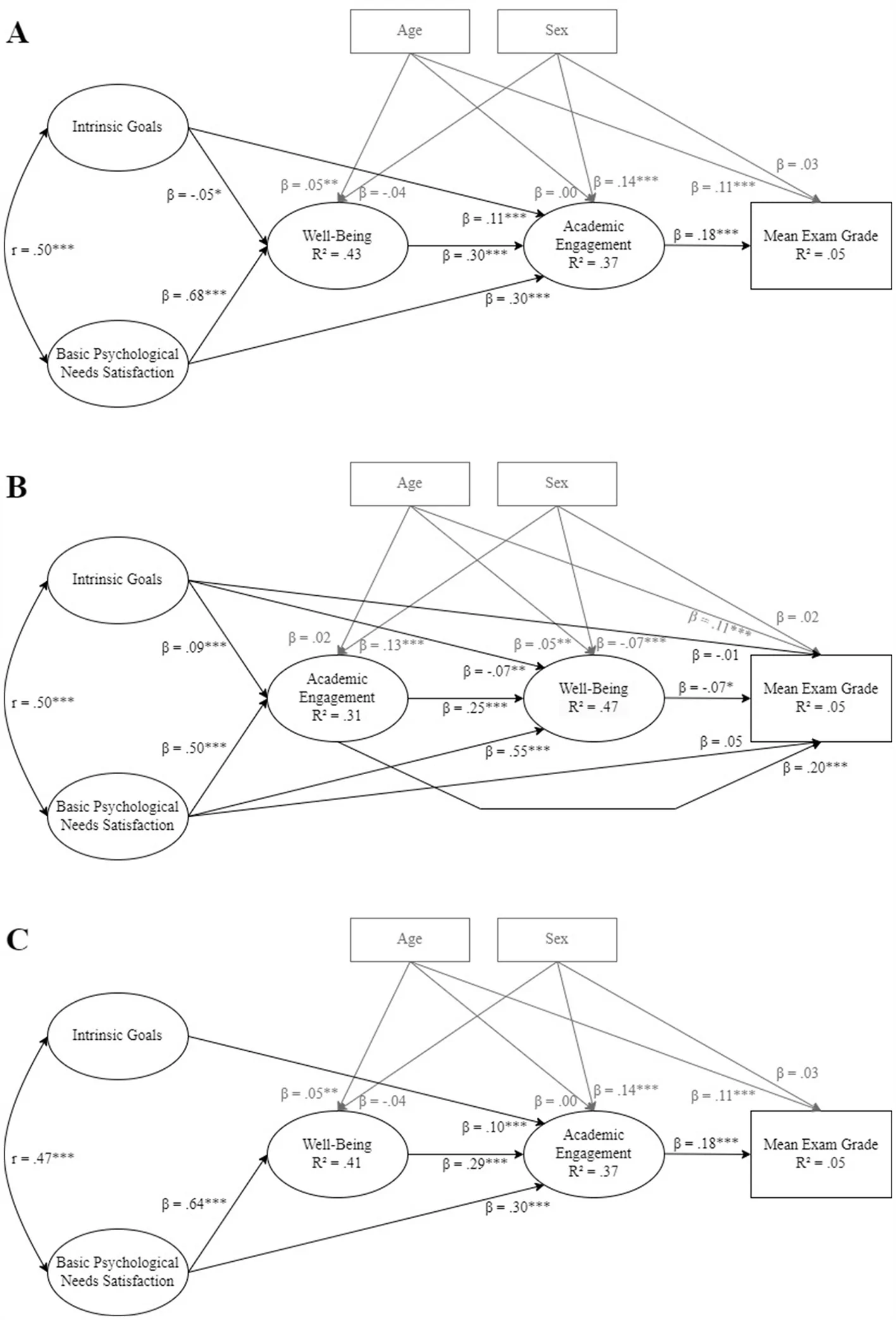
Comparison of the best-supported structural models and the final constrained specification. **(A)** Presents the WB → AE partially mediated model, **(B)** Presents the AE → WB direct-effects model, and **(C)** Presents the follow-up WB → AE constrained model. The model **(C)** was the one finally retained. Values on structural paths represent standardized coefficients (β), whereas the correlation between exogenous variables is reported as Pearson's correlation (*r*). Age and sex were included as control variables and are displayed in gray.

**Table 4:** Indirect paths of the wellbeing → academic engagement constrained model.

Indirect path	*b*	*SE*	*z*	β	95% CI lower	95% CI upper	*p*
BPN-S → WB → AE → Mean exam grade	0.06	0.01	6.63	0.033	0.024	0.043	< 0.001
BPN-S → AE → Mean exam grade	0.09	0.02	6.22	0.054	0.037	0.071	< 0.001
IG → AE → Mean exam grade	0.04	0.01	4.48	0.019	0.010	0.027	< 0.001
WB → AE → Mean exam grade	0.06	0.01	6.54	0.052	0.037	0.068	< 0.001

In the WB → AE constrained model, BPN-S showed a positive indirect association with mean exam grade both through the sequential pathway via WB and AE, β = 0.033, 95% CI [0.024, 0.043], *p* < 0.001, and through AE alone, β = 0.054, 95% CI [0.037, 0.071], *p* < 0.001. IG was also indirectly associated with mean exam grade via AE, β = 0.019, 95% CI [0.010, 0.027], *p* < 0.001. Finally, WB showed a positive indirect association with mean exam grade through AE, β = 0.052, 95% CI [0.037, 0.068], *p* < 0.001 (Table 4). Overall, the model accounted for 41% of the variance in WB, 37% of the variance in AE, and 5% of the variance in mean exam grade (Figure 3). Overall, these results indicate that AE can represent the main pathway through which BPN-S, IG and WB are associated with academic achievement in the retained model.

## Discussion

The present study examines theoretical models linking basic psychological need satisfaction, intrinsic goals, wellbeing, academic engagement, and academic achievement among Italian university students. Grounded in the SDT (Deci and Ryan, 2000) and within a Positive Psychology perspective (Csikszentmihalyi and Seligman, 2000), we primarily tested whether need satisfaction and intrinsic goals would be associated with students' wellbeing, which in turn would link to greater academic engagement and better academic achievement. To address mixed evidence regarding the relationship between wellbeing and engagement, we also compared an alternative theoretical model reflecting the opposite directional ordering between these two constructs. Overall, the findings support the model specifying wellbeing as antecedent to academic engagement, in which basic psychological need satisfaction is strongly associated with wellbeing, whereas both need satisfaction and intrinsic goals are linked to greater academic engagement. Academic engagement emerged as the most proximal predictor of academic achievement, mediating the associations between satisfaction of basic needs, intrinsic goals, wellbeing, and mean exam grade. Notably, the direct association between intrinsic goals and wellbeing was not retained in the final model, as it became negligible once basic psychological need satisfaction was considered.

The first key result of this study concerns the central role of basic psychological need satisfaction. In line with SDT, satisfaction of autonomy, competence, and relatedness was strongly associated with wellbeing and also showed indirect links with academic achievement through both wellbeing and academic engagement. Students who felt more autonomous, competent, and connected to others tended to report better wellbeing and to be more strongly engaged in their academic experience. This pattern is consistent with previous literature identifying need satisfaction as a robust predictor of wellbeing and adaptive functioning across life domains, including education (Van den Broeck et al., 2016; Martela et al., 2023; Ryan and Deci, 2017). It is also in line with evidence showing that students who experience their academic environment as more need-supportive tend to report greater wellbeing, stronger engagement, and more adaptive academic functioning (León and Núñez, 2013; Vansteenkiste and Ryan, 2013; Vansteenkiste et al., 2020). In this sense, our findings reinforce the idea that basic psychological need satisfaction represents a core psychological resource in university students, being tied not only to wellbeing but also to the processes sustaining academic performance.

A second key finding concerns the role of intrinsic goals. Intrinsic goals were directly associated with academic engagement, modeled here as a latent construct reflecting persistence in study choice, university value and sense of belonging, and perceived course value. Moreover, intrinsic goals were indirectly associated with academic achievement through academic engagement. This may suggest that intrinsic aspirations can support students' investment in academic life by providing a sense of personal meaning, fostering different aspects of engagement. Students who prioritize goals such as personal growth, meaningful relationships, and contribution to a broader context may be more likely to perceive their studies as personally significant, to remain committed in the face of difficulties, and to feel more connected to the university environment. This interpretation is consistent with literature suggesting that intrinsic aspirations are positively related to more self-determined forms of motivation and adaptive academic functioning (Bradshaw et al., 2023; Davids et al., 2017).

At the same time, the present findings suggest that the contribution of intrinsic goals to wellbeing can be limited once basic psychological need satisfaction is taken into account. Although intrinsic goals showed positive bivariate associations with all dimensions of wellbeing, the path linking intrinsic goals to wellbeing in the partially mediated structural model was negative, albeit extremely small. This pattern can be plausibly interpreted as a suppression effect due to the shared variance between intrinsic goals and need satisfaction, rather than as evidence of a substantive negative association. Consistently, the hierarchical regression analysis showed that the positive association between intrinsic goals and wellbeing disappeared once basic psychological need satisfaction was included in the model. Overall, this aligns with prior evidence suggesting that the relationship between intrinsic aspirations and wellbeing may attenuate when more proximal SDT processes, such as need satisfaction, are considered (Hope et al., 2019). Therefore, students who were more oriented toward personally meaningful and self-congruent goals tended to show greater commitment to their studies, even if this motivational orientation was not independently associated with wellbeing.

Taken together, these findings suggest a partially differentiated role of the two upstream predictors included in the model. Basic psychological need satisfaction appeared to significantly correlate with wellbeing, whereas intrinsic goals appeared to be more linked to motivational investment captured by academic engagement. In this sense, need satisfaction may be understood as a psychological condition potentially supporting students' adjustment and flourishing, whereas intrinsic goals may operate more as an orienting framework helping students to invest meaningfully in their academic pathway.

A further important result concerns the role of academic engagement. In the retained model, academic engagement emerged as the most proximal predictor of academic achievement and as the main pathway through which satisfaction of basic needs, intrinsic motivations, and wellbeing were linked to mean exam grade. This is consistent with previous literature showing that engagement is positively associated with academic success and may serve as a mechanism translating psychological functioning and motivational aspirations into adaptive academic outcomes (Richardson et al., 2012; Wong et al., 2024). As already anticipated, engagement was operationalized through dimensions related to students' persistence in their chosen course of study, perceived value of the university experience, and sense of belonging. These components are particularly relevant in higher education, where achievement relies not only on cognitive abilities, but also on students' sustained investment in their studies, perceived fit with the academic environment, and willingness to persist over time. Consistent with previous evidence (Freda et al., 2023), our data suggest that these dimensions can sustain university students' academic achievement.

Finally, although previous research has suggested that wellbeing and engagement may be reciprocally related (e.g., Schnell et al., 2025), the present analyses provided support for the model specifying wellbeing as antecedent to academic engagement, rather than the reverse path. This should not be taken as evidence that the opposite ordering is implausible. Rather, within the set of models examined here, the specification in which wellbeing preceded engagement proved to be more parsimonious than the other, while retaining comparable explanatory power. This finding is theoretically coherent with the idea that students who experience higher emotional, psychological, and social wellbeing may be better positioned to invest energy, meaning, and persistence in their academic lives, which may, in turn, support more successful academic outcomes. At the same time, longitudinal research is needed to determine whether these associations are in the direction found or are, plausibly, reciprocally reinforcing over time.

In conclusion, the present study contributes to a more integrated understanding of how need- and goal-based processes, together with wellbeing and academic engagement, relate to academic achievement in higher education. Previous research has often examined the constructs in isolation or within partially specified frameworks, limiting the understanding of how they can jointly contribute to academic achievement (Douglas et al., 2008; Lei et al., 2022). By empirically testing theoretically grounded models, the present findings suggest that need satisfaction can be closely linked to students' wellbeing, whereas intrinsic goals can be more directly related to academic engagement. Therefore, feeling autonomous, competent, and connected to others may provide an important psychological basis for students‘ wellbeing, while pursuing meaningful personal goals may be especially relevant for sustaining their involvement and commitment in their academic experience. Academic engagement may, in turn, represent a key process through which need satisfaction, intrinsic motivations, and academic engagement translate into academic achievement. Integrating these different dimensions may therefore represent an important step toward a more comprehensive understanding of academic functioning and flourishing in higher education.

From an applied perspective, our results suggest that higher education interventions might benefit from considering the support of basic psychological needs as a relevant component of their strategy. This involves creating learning environments that provide students with meaningful opportunities for choice and self-direction, strengthen their sense of competence, and provide relational contexts that foster belonging and connection with peers and academic staff. Interventions should also promote meaningful engagement with academic activities by helping students recognize the personal relevance and value of their studies, connect academic tasks with their broader goals and values, and maintain commitment to their chosen pathway. These two levels of intervention may be particularly important when considered together, as supporting students' psychological needs may contribute to a more positive and sustainable experience of university life, while fostering meaningful engagement may translate into sustained participation, persistence, and investment in academic activities. Indeed, interventions should move beyond a narrow focus on study skills or performance enhancement alone and instead target the broader motivational and psychological conditions that support students' involvement in higher education. Such integrated efforts may ultimately contribute to students' longer-term personal and professional development.

### Strengths, limitations and future directions

A significant strength of the present study lies in its multicenter design, which involved participants from universities across different geographical areas of Italy. This regional heterogeneity enhances the generalizability of the findings and suggests that the observed associations between motivational, psychological, and academic variables may reflect relatively stable processes within the broader Italian higher education context, rather than patterns specific to a single institution or area. In addition, the large sample size and the inclusion of key sociodemographic covariates, such as age and sex, contribute to the robustness of the findings and might reduce the likelihood that the observed associations are driven by unmodeled demographic differences.

However, several limitations of the study should be acknowledged. First, although alternative directional models were compared, the cross-sectional nature of the data precludes firm conclusions about variable ordering or causality. Reciprocal associations between wellbeing and academic engagement cannot be ruled out and should be tested in longitudinal studies. Moreover, because the alternative SEMs were statistically equivalent, their comparison cannot clearly establish the directionality of the associations. Another potential limitation lies in the relatively modest proportion of variance explained in academic achievement. Although the model explains a substantial proportion of variance in wellbeing and engagement, it explains only 5% of the variance in academic achievement. However, this pattern is consistent with prior literature and reflects the multifactorial nature of academic achievement, which is influenced by a wide range of factors beyond motivational and psychological processes, including prior academic preparation, academic discipline, cognitive abilities, and contextual and institutional characteristics (Richardson et al., 2012; Robbins et al., 2004). Moreover, academic achievement was operationalized through mean exam grades, which may offer only a partial view of broader student academic outcomes (York et al., 2015). In addition, the choice to assess this variable using grade ranges was motivated by several considerations, including the bounded and potentially non-normal distribution of university grades (Arthurs et al., 2019), the need to facilitate completion within a relatively long survey, and the aim of reducing inaccuracies associated with recalling exact grade averages. Future research should consider using continuous and objectively recorded institutional indicators of academic achievement, allowing for a more precise assessment of students' performance.

As another limitation, students from different degree programs were analyzed together, despite evidence that academic demands and wellbeing may vary substantially across fields of study. For example, students enrolled in highly demanding programs, such as medicine, often report higher levels of psychological distress and burnout compared to other student populations (Dyrbye et al., 2006; Rotenstein et al., 2016). Future research should examine whether the observed relationships differ across academic disciplines and training contexts. In addition, the sample was obtained through convenience and snowball sampling and consisted predominantly of women. These characteristics may limit the generalizability of the findings. Although academic engagement was modeled as a multidimensional construct, only a subset of its dimensions was included in the present study. Future research should consider a more comprehensive representation of engagement to better capture its full multidimensional nature and potentially differential associations with wellbeing and academic achievement. Basic psychological need satisfaction was modeled as an overall score and need frustration and extrinsic aspirations were not included in the structural model. Although autonomy, competence, and relatedness are theoretically distinct within SDT and may relate to students' wellbeing and academic functioning through partly different processes, their specific contributions were not examined in order to preserve model parsimony and avoid substantially increasing model complexity. Similarly, the exclusion of need frustration and extrinsic aspirations reflected the decision to maintain a theoretically focused framework centered on adaptive motivational processes (e.g., see Vansteenkiste and Ryan, 2013). These constructs may nevertheless provide additional explanatory value; therefore, they should be further examined in the future.

A further and related limitation concerns the fact that, although the items showed statistically significant loadings and satisfactory composite reliability for both basic psychological need satisfaction and intrinsic goals, the global and residual fit indices did not provide unequivocal support for strict one-dimensionality. The present study retained the established aggregate scores and accounted for their measurement error in the SEMs, but did not compare alternative multidimensional structures. Future research may consider multidimensional representations of these measures and evaluate their replicability in independent samples.

The choice of using sex assigned at birth rather than gender identity as a covariate was methodological rather than theoretical, as the distribution of gender identity in the present sample included several small categories that could not be modeled reliably without collapsing substantively distinct identities. Accordingly, the present analyses do not capture the social and psychological experiences associated with gender identity. Future studies with larger and more diverse samples should examine gender identity directly and investigate whether associations among psychological needs, wellbeing, academic engagement, and achievement differ across gender-diverse groups. Eventually, the negative association between intrinsic goals and wellbeing in the SEM model contradicts both the SDT literature and the positive zero-order correlations reported in the manuscript. Although this result was reasonably attributed to a suppression effect, the explanation remains speculative and alternative interpretations could be further explored in future studies.

### Conclusion

The present study provides an integrated account of how satisfaction of basic psychological needs, intrinsic motivations, academic engagement, and wellbeing can jointly relate to academic achievement in Italian university students. Specifically, our findings support a model in which basic psychological need satisfaction represents relevant psychological conditions associated with wellbeing, intrinsic goals can contribute more specifically to students' academic engagement, and academic engagement potentially acts as the proximal mechanism linking these processes to academic achievement. By distinguishing between the complementary roles of these variables and situating them within a single framework, the findings may inform the design of interventions and institutional policies aimed at fostering students' wellbeing together with their academic achievement.

## Data Availability

The original contributions presented in the study are included in the article/Supplementary material, further inquiries can be directed to the corresponding author.

## Footnotes

1 Of the 16 participants who preferred not to report their sex, had missing data on this variable, or selected the intersex category, three had already been excluded because they had missing data on the exam-grade item or preferred not to respond to it.

2 In the present study, the term multicenter refers to participant recruitment conducted across 10 Italian universities located in different geographical areas of the country.

3 Distributional information for the continuous study variables is reported in Supplementary Table S1.

4 All steps of the hierarchical regression were estimated using the same complete-case sample as the SEMs (*N* = 2821).
