# Supplementary material for: From need satisfaction to academic achievement: pathways through wellbeing and engagement among university students across different Italian institutions
Source: Front Psychol. 2026 Oct 15;17:1937840. doi: 10.3389/fpsyg.2026.1937840 (PMC13716869; doi:10.3389/fpsyg.2026.1937840)
Supplement: Supplementary file 1 [file Supplementary_file_1.docx]

#### **S1. Distributional Characteristics of the Study Variables**

Skewness and kurtosis values for the continuous study variables in the total sample are reported in Table S1.

**Table S1**

*Skewness and Kurtosis of Continuous Study Variables in the Total Sample*

| **Variable** | ***M*** | ***SD*** | **Skewness** | **Kurtosis** |
| --- | --- | --- | --- | --- |
| IG | 4.55 | 0.48 | -1.74 | 8.31 |
| BPN-S | 3.92 | 0.63 | -0.55 | 3.58 |
| AE – Persistence in Study Choice | 3.96 | 0.90 | -0.88 | 3.24 |
| AE – University Value & Belonging | 4.40 | 0.62 | -1.14 | 4.02 |
| AE – University Course Value | 4.28 | 0.72 | -1.17 | 4.09 |
| E-WB | 2.93 | 1.12 | -0.35 | 2.39 |
| S-WB | 1.84 | 1.08 | 0.55 | 2.80 |
| P-WB | 3.06 | 1.11 | -0.29 | 2.32 |

*Note****.*** IG = Intrinsic Goals; BPN-S = Basic Psychological Need Satisfaction; AE = Academic Engagement; E-WB = Emotional Well-Being; S-WB = Social Well-Being; P-WB = Psychological Well-Being.

#### **S2. Measurement models**

Measurement properties were examined at the item level via confirmatory factor analysis (CFA) for two complementary purposes: (a) to evaluate measurement quality in the present sample, and (b) to assess whether the use of aggregated scale scores and higher-order latent constructs in the structural equation models (SEMs) was empirically supported.

Consistent with the Self-Determination Theory framework underlying our theoretical model, we focused on the Intrinsic Goals (IG) subscale of the Aspiration Index (Kasser & Ryan, 1995; Duriez et al., 2007) and on the Basic Psychological Need Satisfaction (BPN-S) subscale of the Basic Psychological Need Satisfaction and Frustration Scale (Chen et al., 2015; Costa et al., 2018). Both IG and BPN-S were modeled as single latent factors at the item level. Concerning Academic Engagement (AE), we focused on three of the six scales from the SInAPSi Academic Engagement Scale (SAES; Freda et al., 2021): (1) perceived ability to persist in the chosen university pathway, (2) perceived value of the university and sense of belonging, and (3) perceived value of the university course. These dimensions were conceptualized as reflecting core motivational and academic components of engagement more proximally linked to academic achievement. The remaining AE scales (engagement with one’s relational network, engagement with peers, and engagement with professors) were expected to primarily capture relational and contextual aspects of engagement that may be less directly linked to academic achievement (Freda et al., 2021). A second-order CFA model was specified to examine whether the three selected dimensions could plausibly be represented as indicators of a higher-order AE construct within the present sample. Well-Being (WB) was also modeled as a second-order construct, reflected by emotional, social, and psychological well-being, as assessed by the Mental Health Continuum–Short Form (MHC-SF; Keyes, 2007). CFA fit indices and model degrees of freedom are reported in Table S2, whereas complete standardized loadings, standardized residual variances or disturbances, and reliability estimates are reported in Table S3. Given the comparatively less satisfactory global fit of the one-factor IG and BPN-S models, standardized residual correlations were additionally examined to assess localized discrepancies between the observed and model-implied association structures. These residual correlation matrices are reported in Tables S4 and S5, respectively.

**Table S2**

*CFA Fit Indices*

| **Model** | ***df*** | **CFI** | **TLI** | **RMSEA** | **SRMR** |
| --- | --- | --- | --- | --- | --- |
| IG (1 factor) | 9 | 0.94 | 0.91 | 0.19 | 0.12 |
| BPN-S (1 factor) | 9 | 0.93 | 0.88 | 0.24 | 0.13 |
| AE (2nd order latent construct) | 116 | 0.99 | 0.99 | 0.07 | 0.05 |
| WB (2nd order latent construct) | 74 | 0.99 | 0.99 | 0.09 | 0.05 |

*Note*. IG = Intrinsic Goals; BPN-S = Basic Psychological Need Satisfaction; AE = Academic Engagement; WB = Well-Being; CFI = Comparative Fit Index; TLI = Tucker–Lewis Index; RMSEA = Root Mean Square Error of Approximation; SRMR = Standardized Root Mean Square Residual. Each CFA was estimated using participants with complete responses to all indicators included in the respective model. Model-specific sample sizes were: IG, *N* = 2889; BPN-S, *N* = 2886; AE, *N* = 2875; and WB, *N* = 2868.

**Table S3**

*Complete Standardized Loadings, Residual Variances, and Reliability Estimates*

| **Construct** | **Indicator** | **Standardized loading** | **Standardized residual variance/**  **disturbance** | **CR** | **AVE** |
| --- | --- | --- | --- | --- | --- |
| IG | IG_item1 | .59 | .65 | .88 | .54 |
|  | IG_item2 | .71 | .50 |  |  |
|  | IG_item3 | .70 | .51 |  |  |
|  | IG_item4 | .83 | .31 |  |  |
|  | IG_item5 | .71 | .50 |  |  |
|  | IG_item6 | .85 | .27 |  |  |
| BPN-S | BPN-S_item1 | .69 | .52 | .83 | .47 |
|  | BPN-S_item2 | .69 | .52 |  |  |
|  | BPN-S_item3 | .45 | .80 |  |  |
|  | BPN-S_item4 | .45 | .80 |  |  |
|  | BPN-S_item5 | .84 | .29 |  |  |
|  | BPN-S_item6 | .85 | .28 |  |  |
| AE – Persistence in Study Choice (AE – PSC) | AE – PSC_item1 | .81 | .35 | .84 | .57 |
|  | AE – PSC_item2 | .77 | .40 |  |  |
|  | AE – PSC_item3 | .72 | .48 |  |  |
|  | AE – PSC_item4 | .70 | .50 |  |  |
| AE – University Value and Belonging (AE – UVB) | AE – UVB_item1 | .83 | .32 | .90 | .60 |
|  | AE – UVB_item2 | .45 | .80 |  |  |
|  | AE – UVB_item3 | .86 | .25 |  |  |
|  | AE – UVB_item4 | .83 | .31 |  |  |
|  | AE – UVB_item5 | .80 | .36 |  |  |
|  | AE – UVB_item6 | .81 | .35 |  |  |
| AE – University Course Value (AE – UCV) | AE – UCV_item1 | .79 | .38 | .95 | .72 |
|  | AE – UCV_item2 | .88 | .23 |  |  |
|  | AE – UCV_item3 | .93 | .14 |  |  |
|  | AE – UCV_item4 | .92 | .16 |  |  |
|  | AE – UCV_item5 | .72 | .48 |  |  |
|  | AE – UCV_item6 | .85 | .27 |  |  |
|  | AE – UCV_item7 | .84 | .30 |  |  |
| AE (second-order construct) | AE – PSC | .83 | .32 | .91 | .77 |
|  | AE – UVB | .95 | .10 |  |  |
|  | AE – UCV | .85 | .28 |  |  |
| E-WB | E-WB_item1 | .77 | .40 | .87 | .70 |
|  | E-WB_item2 | .85 | .28 |  |  |
|  | E-WB_item3 | .88 | .22 |  |  |
| S-WB | S-WB_item1 | .72 | .49 | .85 | .53 |
|  | S-WB_item2 | .71 | .49 |  |  |
|  | S-WB_item3 | .78 | .39 |  |  |
|  | S-WB_item4 | .67 | .56 |  |  |
|  | S-WB_item5 | .77 | .40 |  |  |
| P-WB | P-WB_item1 | .76 | .42 | .89 | .57 |
|  | P-WB_item2 | .79 | .37 |  |  |
|  | P-WB_item3 | .69 | .52 |  |  |
|  | P-WB_item4 | .72 | .48 |  |  |
|  | P-WB_item5 | .71 | .50 |  |  |
|  | P-WB_item6 | .84 | .29 |  |  |
| WB (second-order construct) | E-WB | .91 | .17 | .92 | .79 |
|  | S-WB | .81 | .35 |  |  |
|  | P-WB | .94 | .12 |  |  |

*Note*. IG = Intrinsic Goals; BPN-S = Basic Psychological Need Satisfaction; AE = Academic Engagement; AE – PSC = Academic Engagement – Persistence in Study Choice; AE – UVB = Academic Engagement – University Value and Belonging; AE – UCV = Academic Engagement – University Course Value; WB = Well-Being; E-WB = Emotional Well-Being; S-WB = Social Well-Being; P-WB = Psychological Well-Being; CR = Composite Reliability; AVE = Average Variance Extracted. All freely estimated factor loadings had *p* values < .001. For the second-order constructs, residual values represent the standardized disturbances of the corresponding first-order factors.

Because the one-factor IG and BPN-S models showed comparatively less satisfactory global fit, their complete standardized residual correlation matrices are reported in Tables S4 and S5, respectively.

**Table S4**

*Standardized Residual Correlations for the One-Factor Intrinsic Goals Model*

| **Indicator** | **IG_item1** | **IG_item2** | **IG_item3** | **IG_item4** | **IG_item5** |
| --- | --- | --- | --- | --- | --- |
| **IG_item2** | .06 |  |  |  |  |
| **IG_item3** | −.05 | .22 |  |  |  |
| **IG_item4** | −.07 | −.23 | −.18 |  |  |
| **IG_item5** | .17 | −.04 | −.04 | −.07 |  |
| **IG_item6** | −.10 | −.24 | −.23 | .09 | .00 |

*Note.* IG = Intrinsic Goals.

**Table S5**

*Standardized Residual Correlations for the One-Factor Basic Psychological Need Satisfaction Model*

| **Indicator** | **BPN-S_item1** | **BPN-S_item2** | **BPN-S_item3** | **BPN-S_item4** | **BPN-S_item5** |
| --- | --- | --- | --- | --- | --- |
| **BPN-S_item2** | .16 |  |  |  |  |
| **BPN-S_item3** | −.05 | −.10 |  |  |  |
| **BPN-S_item4** | −.04 | −.05 | .40 |  |  |
| **BPN-S_item5** | −.09 | −.08 | −.15 | −.16 |  |
| **BPN-S_item6** | −.09 | −.07 | −.18 | −.17 | .06 |

*Note.* BPN-S = Basic Psychological Need Satisfaction.

The one-factor IG model yielded mixed evidence of fit. Comparative Fit Index (CFI) and Tucker-Lewis Index (TLI) were acceptable, whereas RMSEA = 0.19 and SRMR = 0.12 indicated that the model did not adequately reproduce all aspects of the item-level association structure. Nevertheless, standardized factor loadings ranged from .59 to .85, indicating moderate to strong associations between items and the latent construct. Composite reliability (CR) was satisfactory, and Average Variance Extracted (AVE) exceeded the conventional .50 threshold, supporting adequate internal consistency and convergent validity. Standardized residual correlations ranged from −.24 to .22 (Table S4), indicating localized discrepancies between some observed and model-implied correlations.

The one-factor BPN-S model showed less satisfactory overall fit (see Table S2). Standardized loadings ranged from .45 to .85. CR was satisfactory, and AVE approached the .50 benchmark. Standardized residual correlations ranged from −.18 to .40 (Table S5), indicating that some item-level associations were not adequately reproduced by the one-factor model. Thus, although the items shared meaningful variance and showed satisfactory composite reliability, the results did not provide unequivocal support for strict one-dimensionality.

The second-order CFA model specified for AE demonstrated excellent CFI and TLI. Standardized loadings for the first-order factors were generally strong, and the three first-order dimensions loaded strongly onto the higher-order factor (standardized loadings = .83-.95). CR was high for each first-order factor and for the second-order construct, with AVE values exceeding .50. The second-order construct modeled for WB also demonstrated excellent CFI and TLI. Standardized loadings for first-order factors ranged from .67 to .88. The three dimensions loaded strongly onto the higher-order WB construct (standardized loadings = .81-.94). CR was high for each first-order dimension. For the second-order construct, AVE values were above .50.

Overall, the item-level analyses showed that the indicators loaded significantly on their intended constructs and generally demonstrated satisfactory composite reliability. However, the global and residual fit indices for the one-factor IG and BPN-S models did not provide strong support for their unidimensionality. Nonetheless, IG and BPN-S were retained as aggregated scale scores because this scoring was consistent with the established use of the corresponding subscales (Barberis et al., 2024; Chen et al., 2015; Costa et al., 2018; Duriez et al., 2007; Kasser & Ryan, 1996), and a comprehensive psychometric validation or comparison of alternative factor structures would go beyond the scope of the present study. In the SEMs, these aggregated scores were used as indicators of single-indicator latent variables, with their residual variances fixed on the basis of the corresponding composite reliability estimates to account for measurement error.

**S3. Structural models: rationale and model families**

This section reports the complete structural specifications, model fit indices, explained variance, and comparisons among the progressively constrained models examined within the primary WB→AE and the alternative AE→WB specifications.

Two structural model specifications of structural equation models (SEMs) were estimated to examine the structural relations among Intrinsic Goals (IG), Basic Psychological Need Satisfaction (BPN-S) , Well-Being (WB), Academic Engagement (AE), and academic achievement (mean exam grade coded as a four-level ordinal variable). The two specifications differed in the assumptions about the ordering of WB and AE (WB→AE vs AE→WB).

In the WB→AE specification, IG and PBN-S were specified as predictors of WB, WB was specified as a predictor of AE, and AE was specified as the proximal predictor of mean exam grade.

In the AE→WB specification, IG and PBN-S predicted AE, AE predicted WB, and WB was specified as the proximal predictor of mean exam grade.

Across all models, IG and PBN-S were modeled as single-indicator latent variables using their respective scale mean scores, with measurement error accounted for using the composite reliability estimates obtained from the item-level CFAs (Supplementary Materials, Section S12). IG and PBN-S were allowed to covary. WB and AE were modeled as latent constructs indicated, respectively, by the emotional, social, and psychological well-being dimensions, and by the persistence in study choice, university value and belonging, and perceived course value dimensions. Age and sex (0 = male; 1 = female) were included as covariates predicting the mediators and the outcome. Within the WB→AE ordering, three progressively more parsimonious specifications were tested by removing selected direct paths between constructs. First, a direct-effects structural model (least constrained model) was estimated. In this specification, IG and PBN-S were specified as predictors of both WB and AE, and all constructs (IG, PBN-S, WB, and AE) were specified as directly associated with mean exam grade. This model allowed simultaneous estimation of both direct and indirect pathways linking motivational and psychological factors to academic achievement. Second, a partially mediated structural model was estimated. In this model, IG and PBN-S were specified as linked to both WB and AE, and AE was specified as the sole direct correlate of mean exam grade. Direct links from IG, PBN-S, and WB to mean exam grade were constrained to zero, so that their association with academic achievement was mediated by AE. Third, a fully mediated structural model (most constrained model) was estimated. In this specification, IG and PBN-S were linked to WB, WB was linked to AE, and AE was linked to mean exam grade. Direct links from IG and PBN-S to AE were constrained to zero, yielding a fully sequential mediation structure. The models were evaluated using a theory-guided, general-to-specific approach. Comparisons between successive models differing only in the imposed path constraints were conducted using the WLSMV scaled-shifted chi-square difference test (Rosseel, 2012; Satorra, 2000), together with global fit indices, explained variance, parsimony, and theoretical interpretability (Kline, 2023; West et al., 2012). Model fit indices, explained variance, and scaled chi-square difference tests for the WB→AE specification are reported in Tables S6–S8.

An analogous sequence of progressively more parsimonious specifications was estimated within the alternative AE→WB ordering. First, a direct-effects structural model (least constrained model) was estimated in which IG and PBN-S were linked to both AE and WB, and all constructs were specified as directly linked to mean exam grade. Second, a partially mediated structural model was estimated in which IG and PBN-S were linked to both AE and WB, and WB was specified as the sole direct associate of mean exam grade, with direct paths from IG, PBN-S, and AE to mean exam grade constrained to zero. Finally, a fully mediated structural model (most constrained model) was estimated in which IG and PBN-S were linked to AE, AE was linked to WB, and WB was linked to mean exam grade, yielding a fully sequential mediation structure. The same general-to-specific comparison strategy was applied to these models. Model fit indices, explained variance, and scaled chi-square difference tests for the AE→WB specification are reported in Tables S9–S11.

Because the best-fitting models from the WB→AE and AE→WB specifications were not nested with respect to one another, between-specification comparisons were based on descriptive evaluation of global fit, explained variance, parsimony, and theoretical coherence (West et al., 2012). Model fit indices, explained variance for endogenous variables, and nested comparisons for the WB→AE specification are reported in Tables S4-S6. The same information for the AE→WB specification is provided in Tables S7-S9.

**Table S6**

*Well-Being → Academic Engagement Model SEMs: Model Fit Indices*

| **Model** | **df** | **χ²** | **CFI** | **TLI** | **RMSEA** | **SRMR** |
| --- | --- | --- | --- | --- | --- | --- |
| WB → AE direct-effects model | 34 | 147.15 | 0.95 | 0.95 | 0.05 | 0.02 |
| WB → AE partially mediated model | 37 | 152.07 | 0.95 | 0.96 | 0.05 | 0.02 |
| WB → AE fully mediated model | 39 | 332.25 | 0.90 | 0.90 | 0.07 | 0.05 |

*Note***.** CFI = Comparative Fit Index; TLI = Tucker–Lewis Index; RMSEA = Root Mean Square Error of Approximation; SRMR = Standardized Root Mean Square Residual. Reported CFI/TLI/RMSEA correspond to the robust values provided under WLSMV estimation.

**Table S7**

*Well-Being → Academic Engagement SEMs: Explained Variance for Endogenous Variables*

| **Endogenous variable** | **Direct-effects model R²** | **Partially mediated model R²** | **Fully mediated model R²** |
| --- | --- | --- | --- |
| Well-Being | 0.43 | 0.43 | 0.60 |
| Academic Engagement | 0.37 | 0.37 | 0.41 |
| Mean exam grade | 0.05 | 0.05 | 0.05 |

*Note***.** R² values refer to the proportion of variance explained in each endogenous construct/outcome.

**Table S8**

*Well-Being → Academic Engagement SEMs: Nested Model Comparisons*

| **Comparison** | **Δdf** | **Δχ²** | **p** |
| --- | --- | --- | --- |
| Direct-effects Model vs Partially mediated model | 3 | 5.22 | .156 |
| Partially mediated model vs Fully Mediated Model | 2 | 221.64 | < .001 |

*Note***.** Nested model comparisons were conducted using the WLSMV scaled (shifted) chi-square difference test.

Within the WB→AE SEM specification, the direct-effects model and the partially mediated model showed both good-to-excellent and highly comparable global fit, whereas the fully mediated model fit the data substantially worse (Table S6). Regarding explained variance (Table S7), moving from the direct-effects model to the partially mediated model did not meaningfully alter the proportion of variance explained in the endogenous constructs. Both models accounted for approximately 43% of the variance in WB, 37% in AE, and 5% in mean exam grade. Thus, removing the direct predictors of the outcome did not reduce explanatory power at the level of mean exam grade. By contrast, further constraining the model to impose full serial mediation altered the internal structural relations and was associated with increased explained variance in WB and AE, but at the cost of a marked deterioration in global model fit (Table S6). Nested model comparisons (Table S8) further indicated that constraining to zero the direct paths to the outcome did not significantly worsen fit relative to the direct-effects model, Δχ²(3) = 5.22, p = .156. In contrast, imposing full serial mediation resulted in a substantial decrement in fit relative to the partially mediated model, Δχ²(2) = 221.64, p < .001. Taken together, converging evidence from global fit indices, explained variance, and nested comparisons supported selecting the partially mediated model as the best-fitting and most parsimonious specification within the WB→AE family.

Within the AE→WB specification, the direct-effects model showed good-to-excellent global fit, the partially mediated model showed slightly lower but still acceptable fit indices, and the fully mediated model fitted the data substantially poorer (see Table S9).

**Table S9**

*Academic Engagement → Well-Being SEMs: Model Fit Indices*

| **Model** | **df** | **χ²** | **CFI** | **TLI** | **RMSEA** | **SRMR** |
| --- | --- | --- | --- | --- | --- | --- |
| AE → WB direct-effects model | 34 | 147.15 | .95 | .95 | .05 | .02 |
| AE → WB partially mediated model | 37 | 241.76 | .92 | .93 | .06 | .04 |
| AE → WB fully mediated model | 39 | 521.57 | .83 | .85 | .09 | .06 |

*Note.* CFI = Comparative Fit Index; TLI = Tucker–Lewis Index; RMSEA = Root Mean Square Error of Approximation; SRMR = Standardized Root Mean Square Residual. Reported CFI/TLI/RMSEA correspond to the robust values provided under WLSMV estimation.

Considering the explained variance of endogenous variables (Table S10), the direct-effects model explained approximately 31% of the variance in AE, 47% in WB, and 5% in mean exam grade. The partially mediated model showed comparable explained variance in AE (31%), similar in WB (49%), but lower in mean exam grade (3%). The fully mediated model increased the proportion of variance explained in AE (53%), but at the cost of substantially poorer global fit and without meaningful improvement in outcome prediction.

**Table S10**

*Academic Engagement → Well-Being SEMs: Explained Variance of Endogenous Variables*

| **Endogenous Variable** | **Direct-effects model R²** | **Partially mediated model R²** | **Fully mediated model R²** |
| --- | --- | --- | --- |
| Academic Engagement | .31 | .31 | .53 |
| Well-Being | .47 | .49 | .46 |
| Mean exam grade | .05 | .03 | .04 |

Nested comparisons (Table S11) additionally indicated that constraining to zero the direct paths from IG and BPN-S to mean exam grade (direct-effects model vs. partially mediated model) resulted in a significant deterioration in model fit, Δχ²(3) = 96.24, p < .001. Further constraining the model to impose full serial mediation (partially mediated model vs. fully mediated model) also led to a substantial decrement in fit, Δχ²(2) = 355.34, p < .001. Overall, the pattern of results across model fit indices, explained variance, and nested comparisons indicated that the direct-effects model provided the most adequate representation of the data within the AE→WB specification.

**Table S11**

*Academic Engagement → Well-Being SEMs: Nested Model Comparisons*

| **Comparison** | **Δdf** | **Δχ²** | **p** |
| --- | --- | --- | --- |
| Direct-effects Model vs Partially mediated model | 3 | 96.24 | < .001 |
| Partially mediated model vs Fully Mediated Model | 2 | 355.34 | < .001 |

Finally, moving to the descriptive comparison of the best-supported models from the two SEM specifications, the partially mediated WB→AE model and the direct-effects AE→WB model showed comparably acceptable global fit (see Tables S6 and S9). However, the partially mediated WB→AE model represented a more parsimonious structural specification and achieved comparable explanatory power for mean exam grade without requiring additional direct paths. This model was also broadly consistent with theoretical expectations derived from Self-Determination Theory and prior empirical findings. In light of considerations of parsimony and theoretical interpretability, the WB→AE family was retained as the primary structural framework for interpretation in the main text.

**S4. Items and dimensions of the psychological variables of the study**

This section reports the complete list of items and response scales adopted in the study.

**Tale S12**

*Items and dimensions of the psychological variables of the study*

| **Measure** | **Item** |
| --- | --- |
| **Basic Psychological Need Satisfaction and Frustration Scale (BPNSFS)** | 1. Sento un senso di possibilità di scelta e di libertà nelle cose in cui mi impegno. |
|  | 2. Sento che le mie decisioni rispecchiano quello che voglio veramente. |
|  | 3. La maggior parte delle cose che faccio, le faccio perché “le devo fare”. |
|  | 4. Mi sento costrett* a fare molte cose che io non avrei scelto di fare. |
|  | 5. Mi sento legat* alle persone che si prendono cura di me e alle quali tengo. |
|  | 6. Provo un sentimento di calore verso le persone con cui passo il mio tempo. |
|  | 7. Mi sento esclus* dal gruppo a cui voglio appartenere. |
|  | 8. Sento che le persone che sono importanti per me sono fredde e distanti nei miei confronti. |
|  | 9. Mi sento fiducios* di poter fare le cose bene. |
|  | 10. Mi sento competente per raggiungere i miei obiettivi. |
|  | 11. Mi sento delus* da molte delle mie prestazioni. |
|  | 12. Mi sento insicur* delle mie capacità. |
| **Aspiration Index (AI)** | 1. Sviluppare i miei talenti e la mia personalità. |
|  | 2. Sviluppare amicizie buone e strette. |
|  | 3. Avere successo finanziario nella vita. |
|  | 4. Essere riconosciut* e ammirat* per le cose che faccio. |
|  | 5. Essere attraente e bell*. |
|  | 6. Essere circondat* da amic* che mi vogliono bene. |
|  | 7. Fare qualcosa per contribuire a migliorare la società. |
|  | 8. Essere ricc* e avere beni costosi. |
|  | 9. Essere conosciut* da molti ed essere popolare. |
|  | 10. Essere al passo con le tendenze della moda (abbigliamento, acconciatura dei capelli, ecc.). |
|  | 11. Migliorare me stess* come persona e continuare a crescere. |
|  | 12. Fare piccole cose che contribuiscano a rendere il mondo migliore. |
| **SInAPSi Academic Engagement Scale (SAES)** | 1. Farei meglio a fare altre cose invece che andare all’università. |
|  | 2. Frequentare l'università è per me una grande opportunità. |
|  | 3. Ho buoni rapporti con i/le colleghi/e universitari/e. |
|  | 4. Ho creato delle amicizie significative con alcuni/e colleghi/e universitari/e. |
|  | 5. I/Le docenti chiariscono cosa si aspettano da noi studenti/studentesse. |
|  | 6. I/Le docenti sono di solito disponibili a discutere il mio lavoro. |
|  | 7. I/Le miei/mie docenti mi rispettano come persona. |
|  | 8. I/Le miei/mie docenti sono interessati/e alle mie opinioni e a quello che dico. |
|  | 9. Il corso di studi che sto frequentando è funzionale al raggiungimento dei miei obiettivi professionali. |
|  | 10. Il corso di studi che sto frequentando è un'opportunità per me. |
|  | 11. Il corso di studi che sto frequentando mi interessa. |
|  | 12. Il corso di studi che sto frequentando mi piace. |
|  | 13. Io affronto con serietà la formazione universitaria. |
|  | 14. Io credo che l'università sia un bene per me. |
|  | 15. Io mi confronto con la mia famiglia sul mio percorso universitario. |
|  | 16. Io parlo dei miei progetti professionali con i/le miei/mie amici/amiche. |
|  | 17. Io parlo dei miei progetti professionali con la mia famiglia. |
|  | 18. L'impegno universitario è parte dei miei progetti di vita. |
|  | 19. L'università ha una grande importanza nella mia vita. |
|  | 20. Lascerei subito l'università se avessi una alternativa. |
|  | 21. Mi piace incontrare gli/le amici/amiche all'università. |
|  | 22. Mi sento parte di un gruppo di amici/amiche all'università. |
|  | 23. Nonostante le difficoltà che talvolta incontro nella vita universitaria penso che valga la pena proseguire i miei studi. |
|  | 24. Qualche volta penso di lasciare l'università. |
|  | 25. Secondo me l'istruzione universitaria non vale tutto il tempo, il denaro e gli sforzi che mi richiede. |
|  | 26. Sono convint* che la mia decisione di iscrivermi a questo Ateneo sia stata quella giusta per me. |
|  | 27. Sono sicur* che il corso di laurea che sto frequentando sia il posto giusto per me. |
|  | 28. Studiare con gli/le altri/e studenti/studentesse mi è utile. |
|  | 29. Trovo i miei studi molto significativi per i miei progetti professionali. |
| **Mental Health Continuum - Short Form (MHC - SF)** | 1. Ti sei sentit* felice. |
|  | 2. Ti sei sentit* interessat* alla vita. |
|  | 3. Ti sei sentit* soddisfatt*. |
|  | 4. Hai sentito di poter fare qualcosa di importante per la società. |
|  | 5. Ti sei sentit* parte di una comunità (un gruppo o il tuo quartiere). |
|  | 6. Hai sentito che la nostra società sta diventando un posto migliore per gente come te. |
|  | 7. Hai sentito che le persone sono fondamentalmente buone. |
|  | 8. Hai sentito che il modo in cui funziona la nostra società ha un senso per te. |
|  | 9. Hai sentito che ti piacciono la maggior parte degli aspetti della tua personalità. |
|  | 10. Ti sei sentit* bene nel gestire le responsabilità della tua vita. |
|  | 11. Hai sentito di avere delle relazioni sincere e cordiali con l* altr*. |
|  | 12. Hai sentito di aver avuto delle esperienze che ti hanno aiutat* a crescere e a diventare una persona migliore. |
|  | 13. Ti sei sentit* capace di pensare o esprimere le tue idee e opinioni. |
|  | 14. Hai sentito che la tua vita ha un senso. |

*Note*. The items are presented in Italian (language used). The Basic Psychological Need Satisfaction and Frustration Scale (BPNSFS) and the Aspirations Index (AI) were rated on a 5-point Likert scale ranging from 1 (completely disagree) to 5 (completely agree). The SInAPSi Academic Engagement Scale was rated on a 5-point scale ranging from 1 (not at all) to 5 (completely). The Mental Health Continuum–Short Form (MHC-SF) was rated on a 6-point frequency scale ranging from 1 (never) to 6 (every day).
